# Safety and Efficacy of Acceptance and Commitment Therapy (ACT) in Schizophrenia Spectrum and Other Psychotic Disorders: Protocol for a Systematic Review and Meta-Analysis

**DOI:** 10.3390/mps1040038

**Published:** 2018-10-24

**Authors:** Richard Gray, Stav A. Hillel, Ellie Brown, Amal Al Ghareeb

**Affiliations:** 1 The School of Nursing and Midwifery, La Trobe University, Melbourne 3086, Australia; s.hillel@latrobe.edu.au (S.A.H.); a.zuhairalghareeb@latrobe.edu.au (A.A.G.); 2 Department of Rural Health, The University of South Australia, Adelaide 5001, Australia; 3 School of Health and Human Science, University of Essex, Wivenhoe Park, Colchester CO4 3SQ, UK; 4 Department of Psychiatry, The University of Melbourne, Parkville 3010, Australia; ellie.brown@unimelb.edu.au; 5 IMPACT Strategic Research Centre, School of Medicine, Barwon Health, Deakin University, Geelong 3220, Australia

**Keywords:** acceptance and commitment therapy, cognitive behavioral therapy, psychotherapy, psychosis, schizophrenia

## Abstract

Acceptance and commitment therapy (ACT) has been reported to be effective in the treatment of some psychiatric disorders. It remains uncertain, however, whether ACT is safe and effective in treating schizophrenia spectrum and other psychotic disorders (e.g., psychosis). This protocol describes the methodology for a systematic review and meta-analysis of the safety and efficacy of ACT in the treatment of psychosis. The review will be guided by the standards set by the Cochrane Collaboration. We will search the Allied and Complementary Medicine Database (AMED), Cumulative Index to Nursing and Allied Health Literature (CINAHL), Cochrane Central Register of Controlled Trials (CENTRAL), Excerpta Medica database (EMBASE), EMCARE, Education Resources Information Center (ERIC), MEDLINE, and PsycINFO databases for randomized controlled trials, whose arms are ACT and any comparator, as well as ClinicalTrials.gov, Australian New Zealand Clinical Trials Registry (ANZCTR), and Current Controlled Trials (ISRCTN), for unpublished and ongoing trials. The primary outcome will be any standard (or surrogate) measure of psychotic pathology. The meta-analysis will summarize short-term and long-term effects and different control conditions with or without treatment as usual or comparative to other interventions. In cases where heterogeneity is detected (via *χ*^2^ and *I*^2^), we will adopt the random effects model for computation.

## 1. Background

There is increasing clinical interest in acceptance and commitment therapy (ACT) for the treatment of psychiatric disorders [1]. Philosophically couched in the cognitive behavioral paradigm, ACT is an empirically-based form of psychotherapy that aims to “help…people…live more rewarding lives even in the presence of undesirable thoughts, emotions, and sensations” [2] (p. vii). The therapy claims to have a modest empirical basis and is reported to have been employed successfully in the treatment of affective disorders, eating disorders, and personality disorders [3]. As for disorders on the schizophrenia-psychosis continuum, however, the evidence is seemingly less robust [4].

Globally, approximately 21 to 23 million people have been diagnosed with schizophrenia [5,6]. In England and Wales alone, 300,000 people suffer from the disorder [7], and in Australia, a national survey found a prevalence of 3.1 per 1000 people having a psychotic disorder [8]. The prognosis varies widely from complete recovery to enduring disability [9], and the best available treatments commonly deliver poor outcomes [5]. Antipsychotics, for example, are often poorly tolerated, and even harmful in some instances [10,11]. Economic and social consequences are also observed. It cost the United States approximately $62.7 billion in 2002 [12], and Australia spends $1.38 billion on psychosis annually [13]. At least one factor causing these large figures is the early onset of the disease interfering with the most productive years of life [5]. According to the Schizophrenia Commission, only 8% of those diagnosed with the disorder are in employed work in the United Kingdom [14]. The disorders also continue to bear the stigma that is typically associated with mental illnesses, [15] and according to Catts and McGorry [15], it is the advances in treatment that move us toward stigma amelioration. ACT is a possible new approach in the treatment of schizophrenia spectrum and other psychotic disorders that must be better understood.

## 2. Acceptance and Commitment Therapy and Its Therapeutic Effect on Psychosis

ACT is considered to be part of third wave cognitive behavioral therapy (CBT) [1,2], which is a relatively recent group of therapies that have evolved in an attempt to progress CBT. These therapies remain based on behaviors and cognitions, but acknowledge that challenging and changing these is not always therapeutically beneficial for clients. Instead, the focus is on how a client relates to how they think, feel, or act.

According to O’Donoghue et al., ACT is not symptom-specific [16]; rather, its therapeutic effect can be observed in the upward spiral that ‘psychological flexibility’ facilitates [2]. That is, ACT reduces the epiphenomena of a condition—a clue, perhaps, to account for its purported efficacy in a wide range of both physical and mental diseases [2]. Psychological flexibility refers to a person’s ability to change or persist in a behavior following chosen values and goals [17]. It is obtained by accepting and committing to “negative, irrational, or even psychotic” [17] (p. 9) thoughts. Given that psychological distress ought to be an accepted part of everyday life, ACT prescribes the normal, uninterrupted pursuing of life-fulfilling goals as would be anticipated in a non-clinical population. In other words, ACT does not aim to alleviate symptoms, but is instead aimed at flourishing, as if in the absence of pathology. For psychosis then, ACT might facilitate continued social engagement, sustained enthusiasm for personal interests, and emotional regulation. As Bach notes, it can be changes in behavior—in contrast to positive symptom reduction—that explains betterment in quality of life for people experiencing psychosis [18].

## 3. Rationale

Although systematic reviews and meta-analyses have demonstrated the efficacy of ACT across some mental health disorders, there is still some controversy concerning the quality of the therapy’s evidence base. Atkins et al., for one, point out the differing results of two meta-analyses on the progress of the evidence [19], and others have commented on the possibility of missing trials [20,21,22,23].

Wakefield et al. [24] recently completed a review of quantitative studies that reported on ACT for psychosis. As a result, they did not limit their search to randomized controlled trials (RCTs) and were unable to complete a meta-analysis of the results. Furthermore, there are a number of methodological problems with their review. For one, it seems that a single data set was treated as two, augmenting the pooled sample size. Moreover, the authors did not exclusively measure possible bias in the trials they synthesized (e.g., via the Cochrane risk of bias tool), nor did they attempt to include unpublished data. 

Notably, two systematic reviews have been cited in favor of ACT for psychosis by a practitioner’s manual [16]. One of these was a review by Khoury et al. that measured the efficacy of mindfulness-based therapy (MBT) in a population of psychotic patients [25]. The authors reported the therapy to be moderately effective. While sharing many theoretical bases with ACT, it is unclear why a review focusing on MBT is considered as evidence for ACT in psychosis. Moreover, the same manual cited Cramer et al.’s systematic review of mindfulness- and acceptance-based interventions for psychosis, in which the distinction between MBT and ACT was collapsed. These authors concluded that these interventions can be recommended as an adjunct treatment for patients with psychosis [26]. Unfortunately, neither review attempted to include unpublished data in their syntheses. It is also unclear whether or not harms were reported appropriately.

This current systematic review will synthesize randomized controlled trials of ACT in the treatment of schizophrenia spectrum and other psychotic disorders. This will contribute to the therapy’s empirical claims and illuminate its safety and efficacy as a treatment of the psychoses. According to Division 12 of the American Psychological Association, the efficacy of ACT in psychosis has “modest research support” [4]. Despite this claim, as far as we understand, no systematic review and meta-analysis exclusively concerning ACT in the treatment of psychosis is currently in preparation (the PROSPERO register of systematic review was searched on 21 May 2018).

## 4. Review Question

This review seeks to address the following question: Is acceptance and commitment therapy safe and effective in the treatment of schizophrenia spectrum and other psychotic disorders? ‘Schizophrenia spectrum and other psychotic disorders’ herein refers to the non-affective type. Following recommendations by Schardt et al. [27], our question can be represented schematically as shown in Table 1.

## 5. Methods

A systematic review and meta-analysis of randomized controlled trials of ACT in the treatment of patients diagnosed with any schizophrenia spectrum or other psychotic disorder was conducted. The review will follow the Preferred Reporting Items for Systematic Reviews and Meta-Analyses (PRISMA) Protocols guidelines [28]. See https://figshare.com/s/81071311ad1421711320 for the PRISMA Protocol checklist. The Cochrane Handbook for Systematic Reviews of Interventions [29] will also inform this review. This review was registered on PROSPERO on 21st May 2018 by R.G. (registration number: CRD42018097200).

## 6. Data Sources

### 6.1. Databases

The following databases will be searched for eligible studies: Allied and Complementary Medicine Database (AMED), Cumulative Index to Nursing and Allied Health Literature (CINAHL), Cochrane Central Register of Controlled Trials (CENTRAL), Excerpta Medica database (EMBASE) via Ovid, EMCARE via Ovid, Education Resources Information Center (ERIC), MEDLINE (including ahead of print, in-process, and other non-indexed citations) via Ovid, and PsycINFO via Ovid.

### 6.2. Clinical Trial Websites

We will supplement these sources by searching on ClinicalTrials.gov, Australian New Zealand Clinical Trials Registry (ANZCTR), and Current Controlled Trials (ISRCTN) for unpublished and ongoing trials. This is but one attempt to decrease possible publication bias—a vital step, as there has been a suspicion that many trials have not been synthesized in previous reviews [21,22]. We will search *psychosis* and *schizophrenia* for the condition, and *acceptance and commitment therapy* and *ACT* for intervention.

## 7. Eligibility Criteria

Studies retrieved from databases and clinical trial websites must exhaust the following eligibility criteria to be included in the systematic review and meta-analysis.

### 7.1. Types of Participants

A study is eligible if its sample meets the following criteria:

- is 18+ years old, of any gender;
- has a diagnosis of a schizophrenia spectrum or other psychotic disorder (using any standard diagnostic criteria; e.g., Diagnostic and Statistical Manual of Mental Disorders (DSM)-5, Feighner criteria [30], International Statistical Classification of Diseases and Related Health Problems (ICD)-10) at any stage of illness.

A study is ineligible if its sample meets the following criteria:

- has a schizophrenia spectrum or other psychotic disorder of the affective type (e.g., schizoaffective disorder);
- has a developmental impairment, intellectual disability, or organic psychosis;
- has a primary drug or alcohol addiction.

### 7.2. Types of Studies

A study is eligible if it meets the following criteria:

- is a randomized controlled trial whose arms (at least two) are ACT delivered via any medium (e.g., individual, group, telephone, online) and any comparator(s);
- provides sufficient statistics to be included in the meta-analysis;
- is in English;
- is published between 1999, when Hayes et al. first described ACT [31], and the search date.

A study is ineligible if it meets the following criteria:

- less than half of its sample has a primary diagnosis of a psychotic disorder;
- it reports extensions to previously published trials.

## 8. Search

The search strategy will include the following keywords (italicized) and medical subject headings (MeSH) (in bold) on the databases: *acceptance commitment therapy*, *ACT*, *clinical behavior analysis*; **Cognitive Therapy**/ *psychosis*, *psychotic disorder*, *schizophrenia*; **Psychotic Disorders**/; **Schizophrenia**/. Following recommendations of Wolters Kluwer Health [32], keywords will include special characters in order to capture any orthographic variation. Keywords will be conjoined with MeSH in a highly-sensitive syntax via the Boolean operator OR.

### 8.1. Search Strategy

The complete search strategy is as follows (keywords are italicized; MeSH are in bold; Boolean operators are capitalized; limiting filters are prefixed with L-):

1. ‘*acceptance commitment therapy*’ OR act OR ‘clinic* behavi?r OR analy*’. mp. [mp = title, abstract, original title, name of substance word, subject heading word, keyword heading word, protocol supplementary concept word, rare disease supplementary concept word, unique identifier, synonyms]
2. *psychos?s* OR ‘psychotic disorder*’ OR *schizophrenia*. mp. [mp = title, abstract, original title, name of substance word, subject heading word, keyword heading word, protocol supplementary concept word, rare disease supplementary concept word, unique identifier, synonyms]
3. exp **Cognitive Therapy**/
4. exp **Psychotic Disorders**/ or exp **Schizophrenia**/
5. 1 OR 3
6. 2 OR 4
7. 5 AND 6
8. limit 7 to L-randomized controlled trial

The subject heading **Cognitive Therapy**/ is **Acceptance and Commitment Therapy**/ in PsycINFO. ERIC does not allow the use of subject headings. Accordingly, the search strategy for ERIC is as follows:

(‘*acceptance commitment therapy*’ OR *act* OR ‘clinic* behavi?r analy*’) AND (*psychos?s* OR ‘psychotic disorder*’ OR *schizophrenia*)

### 8.2. Primary Outcome, Timing, and Effect Measures

The review’s primary outcome is any standard measure of psychotic pathology that may include, but is not limited to, the Positive and Negative Syndrome Scale (PANSS), the Brief Psychiatric Rating Scale (BPRS), or the Psychotic Symptom Rating Scales (PSYRATS). However, as the therapeutic mechanism of ACT is not the alleviation of psychotic symptoms, but rather the epiphenomena of psychotic symptoms, it seems rational to anticipate that researchers have used surrogate measures in trials (e.g., rehospitalization). Accordingly, any such surrogate measure will be able to function as a primary outcome in our review. Measurement is from baseline to end of treatment or the nearest available follow-up.

### 8.3. Secondary Outcomes, Timing, and Effect Measures

The review’s secondary outcomes are any validated measures of personal recovery such as the Recovery Style Questionnaire or Harms (any adverse and serious adverse events following Good Clinical Practice (GCP) guidelines). Measurement is from baseline to end of treatment or the nearest available follow-up.

## 9. Data

### 9.1. Data Retrieval

The data retrieved by the database and clinical trial websites searchers will be uploaded to the referencing manager EndNote (https://endnote.com/). EndNote will remove duplicates from the review’s sample. We will make the remaining data publically available on Figshare (https://figshare.com/), an online repository for academic outputs.

### 9.2. Data Screening

R.G. and S.A.H. will independently screen titles and abstracts in Covidence (https://www.covidence.org/). Covidence is a screening software tool developed for systematic reviews. E.B./A.A.G. will resolve any disagreements. R.G. and S.A.H. will independently screen full texts. E.B./A.A.B. will resolve any disagreements. A flow diagram following the PRISMA statement will document the data management.

### 9.3. Data Extraction

A standardized form will be used to extract data for evidence synthesis. R.G. and S.A.H. will extract data. Risk of bias will be determined using the Cochrane risk of bias tool (performance, detection, attrition, reporting, other). Inter-rater reliability will be measured using Cohen’s *κ*. The extracted data will be made publicly available on Figshare. The following data items will be extracted: study citation, disorder of interest, setting, dose of intervention, delivery medium of intervention, primary endpoint, number of participants allocated to intervention and control groups, number of analysed participants allocated to intervention and control groups, calculation of sample size, pre- and post-intervention/control summary statistics, intervention and control harm, quality of blinding, quality of randomization, bias, analysis type, registration status, and country of origin.

## 10. Synthesis

### 10.1. Meta-Analysis

We will undertake meta-analyses for end-of-treatment effects and different control conditions with or without treatment as usual or comparative to other interventions. Meta-analysis will be conducted if at least three RCTs for a specific comparison are available.

### 10.2. Heterogeneity and Modeling

Dependent on summary statistic type, Pearson’s *χ*^2^ will measure heterogeneity due to chance. A *p*-value (0.0000) less than the significance level (0.05) will motivate a random effects model. Higgins’s *I*^2^ will measure heterogeneity due to variance in true effect sizes. Conservatively, less than 25% will motivate a random effects model. Following the Cochrane Handbook of Systematic Reviews of Interventions [29], we will test for funnel plot asymmetry if a minimum of ten studies are included in the meta-analysis.

### 10.3. Summary Statistic and Weights

The weighted average of the most common summary statistic across the eligible studies will be reported. While methods do allow differing summary statistics to be pooled together [33,34,35], it is unclear to what degree this represents a distortion of data. Any uncommon summary statistic will thus receive its own forest plot. In order to account for heterogeneity (determined by *χ*^2^ and *I*^2^), we will assign the following weight to each study: $w_{i}^{*}=\frac{1}{v_{i}+v^{*}}$ (where *v_i_* is within-study variance and v* is between-study variance); alternatively, $w_{i}=\frac{1}{v_{i}}$.

## 11. Conclusions

ACT is employed in the treatment of a number of psychiatric disorders. While treatment outcomes for affective, eating, and personality disorders are said to be positive, there is more uncertainty concerning their efficacy in treating schizophrenia spectrum and other psychotic disorders. This systematic review aims to determine the safety and effectiveness of ACT in psychoses and add to the therapy’s evidence base.

## Figures and Tables

**Table 1:** Review question compartmentalized according to the PICO(R) mnemonic. ACT—acceptance and commitment therapy.

P	Schizophrenia Spectrum/Psychotic Disorders
**I**	ACT
**C**	Ø
**O**	Symptom alleviation
**R**	Randomized controlled trial
